# Reports on Cities, Towns, and Districts

**Published:** 1856-07

**Authors:** 


### SANITARY AND SOCIAL SCIENCE.

### REPORTS OF CITIES, TOWNS, & DISTRICTS.

### ON THE AVERAGE MORTALITY AND PUBLIC HEALTH OF THE HASTINGS DISTRICT.

(*Continued from* vol. i, p. 419.)

In the former paper was given the annual rate of mortality in the Hastings District, and also the number of deaths from the principal diseases occurring in each month and each season. It is interesting and important to consider also the relative proportion which the deaths from different diseases bear, (1) to the total number of deaths from all causes; and (2) especially to the population of the place. It must, however, be borne in mind, that in Hastings the conclusions drawn from the registers of deaths are greatly influenced by the large number of invalids who resort thither during their last illness; a source of error that will be more particularly noticed hereafter.

With respect to the former inquiry, viz. the relative proportion which the deaths from different diseases and classes of diseases in Hastings bear to the total number of deaths from all causes, we need not do more than mention some of the laws laid down by Dr. Farr, and show how far they are confirmed by the experience of this place.

In the First Annual Report of the Registrar-General (p. 111, 8vo.) he says: "Wherever the absolute mortality is low, the number of deaths in the epidemic [*zymotic*] class, is less than the number in the pulmonary class; and, on the contrary, wherever the deaths in the first class exceed or equal those in the third [now the *sixth*, with the addition of *phthisis*], it may be affirmed that the absolute mortality is high." In the Hastings District, the average annual number of deaths in the zymotic class is about 68; the deaths caused by pulmonary diseases, including *phthisis*, are about 102.

Again, in the Thirteenth Report (Appendix, p. 133), he says: "In unhealthy years, and in unhealthy places, it may be laid down as a general law, that the zymotic diseases (Class I) are always more fatal than the diseases in the two next groups (Classes II, III); and that in healthy seasons and places the converse is true." In the Hastings District, the average annual deaths from Classes II and III, amount to about 106; while the deaths from zymotic diseases (as has been mentioned) are only about 68.

*Average Number of Deaths in two Decennial Periods, from different Causes, and Classes of Causes.*

| Causes of Death. | 1838 to 1847. Total number of deaths. | Proportion to 1000 deaths. | Proportion to 10,000 persons living. | 1845 to 1854. Total number of deaths. | Proportion to 1000 deaths. | Proportion to 10,000 persons living. |
|---|---|---|---|---|---|---|
| I. Classes of Disease. | | | | | | |
| Zymotic diseases | 48·5 | 169·9 | 31·6 | 68·3 | 183·0 | 34·9 |
| Dis. of uncertain or variable seat | 16·1 | 56·4 | 10·5 | 19·7 | 52·8 | 10·0 |
| Tubercular diseases | 83·7 | 293·3 | 54·2 | 86·7 | 232·3 | 44·1 |
| Dis. of nervous system | 31·0 | 108·6 | 20·1 | 42·7 | 114·4 | 21·3 |
| Dis. of organs of circulation | 5·7 | 20·0 | 3·5 | 12·7 | 34·0 | 6·3 |
| Dis. of respiratory organs | 19·7 | 69·0 | 12·7 | 39·8 | 106·6 | 19·5 |
| Dis. of digestive organs | 17·2 | 60·3 | 11·2 | 21·8 | 58·4 | 10·9 |
| Dis. of urinary organs; Childbth.& dis. of generative org. | 5·7 | 20·0 | 3·6 | 6·4 | 17·2 | 3·2 |
| Dis. of organs of locomotion; Dis. of integumentary system | ·8 | 2·8 | ·5 | 2·3 | 6·2 | 1·1 |
| Malformation; Infantile debility; Atrophy | 16·9 | 59·2 | 10·9 | 26·4 | 70·7 | 13·3 |
| Senile debility | 25·2 | 88·3 | 16·3 | 30·3 | 81·2 | 15·3 |
| Sudden (causes unascertained) | 1·5 | 5·3 | 1·0 | ·6 | 1·6 | ·3 |
| External causes | 8·6 | 30·1 | 5·5 | 14·8 | 39·7 | 7·6 |
| Causes not specified, etc. | 4·8 | 16·8 | 3·2 | ·8 | 2·1 | ·4 |
| Total | 285·4 | 1000·0 | 184·8 | 373·3 | 1000·2 | 188·2 |
| II. Individual Diseases. | | | | | | |
| Small-pox | 2·6 | 9·1 | 1·8 | 2·5 | 6.7 | 1·3 |
| Measles | 9·7 | 34·0 | 6·5 | 7·6 | 20·1 | 4·1 |
| Scarlatina | 5·3 | 18·6 | 3·6 | 2·0 | 5·4 | ·7 |
| Hooping-cough | 8·8 | 30·8 | 5·6 | 10·9 | 29·2 | 5·5 |
| Diarrhœa | 5·3 | 18·6 | 3·3 | 12·5 | 33·5 | 6·3 |
| Fever | 5·2 | 18·2 | 3·4 | 10·9 | 29·2 | 5·5 |
| Dropsy | 10·2 | 35·8 | 6·7 | 8·7 | 23·3 | 4·5 |
| Phthisis | 62·4 | 218.7 | 40·4 | 61·9 | 165·9 | 31·5 |
| Hydrocephalus | 16·3 | 57·1 | 10·7 | 11·0 | 29·5 | 5·6 |
| Apoplexy | 8·5 | 29·8 | 5·5 | 13·3 | 35·6 | 6·7 |
| Paralysis | 3·8 | 13·3 | 2·4 | 5·3 | 14·2 | 2·7 |
| Convulsions | 14·0 | 49·1 | 9·2 | 15·2 | 40·7 | 7·6 |
| Disease of heart | 5·2 | 18·2 | 3·2 | 11·8 | 31·6 | 5·9 |
| Bronchitis | 2·0 | 7·0 | 1·2 | 13·5 | 36·2 | 6·5 |
| Pneumonia | 10·9 | 38·2 | 7·0 | 18·7 | 50·1 | 9·2 |

Once more, in the First Annual Report (p. 116) he says: "It may be laid down as a general principle, that whenever the proportion of deaths from phthisis, compared with the total deaths, is high, the absolute mortality is low, and that the absolute mortality from phthisis itself is low. . . . The deaths out of the living express the real tendency to phthisis." In the Hastings district, the deaths attributed to phthisis in the ten years, 1845-54, were about sixty-two annually, or more than 16½ per cent. of the total deaths, a high proportion compared with the whole of England; and therefore, as the average mortality of Hastings is low, agreeing with the former part of Dr. Farr's law. To the latter clause, which requires "the absolute mortality from phthisis itself to be low", Hastings forms an apparent exception; for it appears that in the same period 31½ out of every thousand of the population died of phthisis annually, which is a larger proportion than is carried off by this disease throughout the whole of England. The exception, however, to Dr. Farr's rule is probably only apparent, and admits of an easy explanation, when it is borne in mind that the deaths from phthisis in Hastings are swelled enormously by the large number of strangers who die of this disease, which, in fact, constitutes more than one-third of the whole.* But the deaths occurring among strangers will be specially noticed below. It will be seen that each of Dr. Farr's laws rests upon the same two facts, viz., 1, that the number of deaths from phthisis, by far the most fatal disease in his catalogue, varies comparatively little in different seasons and localities; and, 2, that in unhealthy years and places, the excessive mortality is caused principally by the deaths in the zymotic class. These facts furnish the solution to the third of his statements mentioned above, which is expressed almost like a paradox or riddle. The inquiry into the actual number of persons that are annually carried off by different diseases and classes of diseases is more important.

The deaths occasioned by zymotic diseases in the ten years 1838-47 was 3·16 per 1,000 annually, and in the ten years 1845-54 they were 3·49 per 1,000. The lowest mortality from this class was 1·46 in 1844; the highest was 8·18 in 1849, in which year the cholera carried off many labourers who were then engaged in making a railway at Hastings. This rate of mortality is low when compared with all England, and still more so when compared with the generality of *town* districts. The slight increase in the rate of mortality in the *second* decennial period is probably due entirely to the cholera in 1849, in which year the deaths from this disease amounted to 2·66 per 1,000. Of the most fatal diseases of this class, the mortality from small-pox, scarlatina, diar-

* *Total* average deaths from phthisis, about 62; average deaths from phthisis among *strangers*, about 24.

rhœa, and fever, is low; that from measles and hooping-cough is rather high. Small-pox was epidemic in 1839 and 1850; during nine consecutive years (1840-48) no death was attributed to this disease. Measles was epidemic in 1838, 1841, 1845, 1849, and 1850; scarlatina in 1839 and 1840; hooping-cough in 1839, 1843, 1847, 1849, 1850, 1853, and 1854. Two diseases in the zymotic class were more fatal in the second decennial period than in the first, viz., diarrhœa and fever (including under this name all the common continued fevers of this country*). A similar increase of diarrhœa has been noticed throughout all England. The only known cause for the greater tendency to fever is the rapid increase of population without a corresponding improvement in sanitary arrangements. Probably almost all the fatal cases referred to the zymotic class originated in Hastings; but with respect to small-pox, measles, scarlatina, and hooping-cough, it is so common for invalids to come to this place when just recovering from these diseases and still capable of infecting others, that it is rather a matter of surprise that the town should ever be free from them.

The second class (diseases of uncertain or variable seat) occasions about one death annually among every thousand of the inhabitants—a proportion which differs very slightly from the average mortality throughout England. The only name in this class that deserves special notice is "dropsy," to which more deaths are attributed in the first decennial period than in the second. This would seem at first sight to imply a decrease either in the frequency of the disease, or at least in its severity; but it does in fact only indicate an improvement in pathological science, and greater care in registering the causes of death; many deaths, which would formerly have been attributed to "dropsy," are now more correctly assigned to disease of heart, liver, kidneys, etc.

The fourth, or tubercular class, is the most numerous of all; and, next to the zymotic, the most important. The proportion of deaths attributed to this class in the first decennial period was annually 5·42 per 1,000, in the second it was 4·41; but, as in the case of dropsy, just mentioned, this does not indicate any diminution in the frequency of these dis-

* Dr. Mackness (*Hastings Considered as a Resort for Invalids*, p. 44) has overlooked this sense of the word "*typhus*" in the Reports of the Registrar-general, and has therefore fallen into serious error by affirming that "this fatal and dreadful malady has been about nine times less frequent at Hastings than the usual average in other parts of England". It is true that the mortality from fever (or *typhus*) is comparatively low in Hastings, but not by any means to the extent stated by Dr. Mackness.

eases, or any improvement in our mode of treating them. The decrease arises entirely from the vagueness and ambiguity of the word "*consumption*," which term is now less frequently used than formerly, and is employed in a more definite and scientific sense. The deaths assigned to phthisis in the last ten years amount annually to 3·15 per 1,000, which is a high rate of mortality, attributable (as has been said above), in a great degree, if not entirely, to the number of strangers who die in Hastings of this disease. It would be very desirable to know the proportion of *bonâ fide* inhabitants who are carried off by phthisis; but this it is impossible to discover, as we have no means of knowing how many of the persons enumerated at the last census were visitors, and how many were residents. This difficulty applies to all places much frequented by visitors; but there is another, which is peculiar to Hastings, Torquay, and other similar localities, which is fairly stated by Dr. Mackness, viz., the numerous individuals "who, having resided several years at Hastings on account of impaired health, are considered as inhabitants, but who originally came from other places, with a constitution highly predisposed to tubercular disease" (p. 68). The highest rate of mortality from phthisis during the last seventeen years was 5·20 per 1,000 in 1843; the lowest was 2·54 per 1,000 in 1853; but it must be borne in mind that the former number is undoubtedly exaggerated.

Hydrocephalus (which, though classed exclusively among the tubercular diseases, certainly comprehends many cases of simple effusion after inflammation) appears to be somewhat more fatal in Hastings than in all England, though probably not more than in other towns. It would also seem to have been more fatal in the first decennial period than in the second; but this diminution of numbers is due to the improvement of registration effected by the introduction of the forms for medical certificates in 1845, as is the case with dropsy, phthisis, and some other diseases.

The deaths from diseases of the nervous system are comparatively less than throughout all England. There is a great difference in the number of deaths caused respectively by apoplexy and by paralysis, which, throughout the whole of England, and also in the several registration divisions, are nearly equal: possibly this discrepancy (if not accidental) may depend partly on the medical certificates, and partly on the classification of the certificates.

"Convulsions" is rather a vague term as a cause of death even now, and was formerly used still more indefinitely:

accordingly, we find that the proportion of deaths attributed to this disease in the first decennial period was higher than in the second. The number of children carried off by convulsions in Hastings appears to be considerably less in proportion than throughout all England,—a fact of some importance, when we remember that this is one of the diseases especially influenced by dirt and wretchedness.

The deaths from disease of the heart, etc., are (comparatively) rather less numerous than throughout all England. They have greatly increased in number in the second decennial period, but this is merely owing to the greater facility with which disease of the heart is now distinguished: no doubt, many of the deaths that would formerly have been attributed to *dropsy* are now entered under *disease of heart.*

The deaths from diseases of the respiratory organs have increased during the latter decennial period, but are less numerous than throughout all England; as is also the case with each of the principal diseases, bronchitis and pneumonia. It may be noticed (what perhaps would hardly have been expected) that among those who die of these diseases in Hastings the number of strangers is comparatively small.

The deaths from diseases of the digestive organs were nearly equally numerous in each decennial period, and rather less numerous than throughout all England. None of these diseases require any special notice, nor do any of the other classes in the statistical nosology, except that the deaths attributed to old age have been gradually becoming less numerous,—another fact indicating improved skill in detecting disease, and greater care in registering the true cause of death.

---

### REPORT ON THE SANITARY STATE OF THE CITY AND BOROUGH OF CANTERBURY, PARTICULARLY DURING THE YEARS 1854 AND 1855.

By GEORGE RIGDEN, M.R.C.S., Surgeon to the Canterbury Dispensary.

THE city of Canterbury, the capital town of East Kent, is situated in latitude 51° 17′ north, and longitude 1° 4′ east, and is the centre of a large and extremely fertile district, producing principally hops and grain, which give employment to a numerous and generally healthy peasantry. The city is noted for its beautiful cathedral, and for its numerous remains of the ancient British, Roman, and medieval ages. It has three markets in the week, which are well supplied with live stock, vegetables, and grain. Although within the last century it has been the seat of a numerous silk weaving population, this branch of industry has been removed to other and distant localities, and it can scarcely now be said to have much pretension to be ranked as a manufacturing city.

*Geology.* Canterbury is situated in a valley on the river Stour, which passes through it, for the most part, from the south-west to the north-east direction; this river rises above Ashford, and after the addition of many springs to its stream becomes a goodly river. On its approach to Canterbury, it branches off into two streams, the one by Westgate to Dean's Mill, and thence to a point below Barton Mill. The other portion of the stream passes through the centre of the city near East Bridge Hospital, where it is crossed by King's Bridge, and continues its course to supply the Abbot's Mill, immediately below which it has a connecting branch with the other stream; after which it continues to Barton Mill, and shortly after unites with that branch which passes under Westgate, and in one stream glides on to Sturry. It will thus be seen that one part of the city is an island. At one time this river must have been a magnificent one, extending as it did from what are now hills on either side, and varying from one to two miles across.

Standing upon the left hand side of the bank at Whitehall, about a mile above Canterbury, it is at once seen that this river has found its passage through the chalk, the extreme limit of whose range is well seen at this point on the left side; but in a line from thence to the foot of the hill, called St. Thomas's hill, and to St. Stephen's, chalk is found under the alluvial. Thus, at St. Dunstan's cutting for the railway, chalk was found, as also at the brewery of the Messrs. Flint in St. Dunstan's, under the bed of the river at several points, both right and left of the city towards Sturry. On the right bank or side of the city the chalk rises into hills of considerable altitude, which attain their summit beyond Nackington, on a road leading to Swadling and just beyond Heppington, where is a circular mound of trees, visible at many points near the western and northern portions of Canterbury. The other side of this mound passes rapidly into a valley, through which the Nailbourn and lesser Stour pass, and, finding their way to Bridge, Littlebourn, and Ickham, finally join the Canterbury river; the united waters of these streams enter the sea at the Sandwich haven. Still remaining at the same place, the left hand side of the bank at Whitehall, a more remarkable feature presents itself; namely, that on the left hand. As soon as the chalk disappears, we meet with the London clay, of which the high ground of St. Thomas's Hill, St. Stephens, Hale's Place estate, forms the meeting of the chalk and London clay, whilst on the right of the city we have alluvium covering the chalk; then the grand mass before described, but also a series of clays, gravels, and sands, that belong to the so-called plastic clay, and which conceal the chalk from view. Thus, St. Martin's Hill, the barracks, and passing down to the right of the stream towards Sturry and Fordwich, are all on the plastic, while, as before stated, the opposite bank on the left is wholly on the London clay. The proof of this is that, at the digging for the Whitstable tunnel, after passing a bed of sand, the clay with its corresponding fossils, lignite, and gypsum, were abundantly found. The same has recently occurred in the sinking of a well at the Clergy's Orphan Asylum, St. Thomas's Hill, where, after piercing through three feet of gravel, nine feet of yellow clay, with indurated nodules of clay interspersed, forty-seven feet of blue clay, with septaria, seven feet of imperfect clay stone, seventy-four feet of sand, and five feet of fossil bed, consisting of sand with venericardia, etc., water was abundantly procured, which rises in the well to within one hundred and twenty-two feet six inches from the surface. Thus it will be seen that the river Stour at Canterbury passes through a gorge across the main line of the chalk hills, for the highest point of the chalk is on the left hand as we advance up the river. The elevation of the chalk must have taken place before the river flowed in its present bed, for the stream could never have forced its passage through a range of chalk hills from one to two hundred feet high; indeed, upon examining the inclination of the strata, it is evident that, at some prior period, the chalk has been upheaved, which leaving other portions depressed, has allowed the water to find its course between its principal altitudes. Canterbury is therefore situated upon the chalk near the edge of the London basin, and also in part upon the beds of the plastic clay, whilst the whole of the centre is placed upon alluvial or modern *débris*.

*Elevation.* The city is reported to be from twelve or fifteen feet at its lowest, to about forty feet at its highest level above that of the sea, and there is no difficulty in procuring water by wells pierced into the chalk at any part of its area.

*Water Supply.* The city is supplied with water from the river generally, but also in part from the Water Company, who pump it from the river above the town into tanks at the top of the old Norman castle, where it is filtered and supplied to the tops of most of the houses. The water from the north-west, that is collected by the drainage from the table-land of the London clay, is mostly allowed to run to waste ; but that on the south-east, collected from the plastic, is most excellent, and furnishes a constant supply to taps in the chief thoroughfares in the city. On the two sides of St. Martin's Hill are reservoirs ; those on the south supply the city generally; those on the north afford a supply to the cathedral precincts. In addition to these public works, many of the houses have wells attached to them, which, being pierced into the chalk, afford an ample supply of water, excellent to the palate, but containing considerable portions of lime, which is freely deposited upon the base and sides of the vessels in which it is boiled ; this is not the case with the soft water supplied from the reservoirs on St. Martin's Hill.

In a city so long inhabited as Canterbury, it may be imagined that the accumulated matter of centuries is not a pure stratum through which to obtain a good wholesome water; and if such should find passage into the good water from the chalk wells, the water becomes deteriorated, and even the cause of disease. Such cases have occurred in Best Lane, Burgate Lane, and in Cotton Mill Lane, within a few years.

*Drainage.* It is to be regretted that greater advantage has not been taken of the natural inclination of the surface upon which Canterbury is situate to effect a good system of drainage. Although with but few exceptions its streets are supplied with underground drains, generally of sufficient calibre, these have unfortunately been constructed at different periods and upon no systematic plan; they are therefore placed upon various and uncertain levels, and thus in many places produce impediments to the regular flow of refuse liquids, and often emit, particularly at the numerous cesspools, most offensive and even poisonous gases.

*Houses and Streets.* The houses, for the most part built of brick, and the streets, are generally well adapted to the wants of the population. The higher class houses, situated on the more rising ground, are also well ventilated and dry; but the abodes of the poor are but cottages, and are generally situated upon the lowest level of the city, where the ground is at all times damp, not only from the nature of the soil, but from the occasional overflowing of the river, produced in a great measure by the large corn-mills impeding the natural flow of its waters, and thus causing the saturation of the earth. Hence arise much misery and disease among the population who inhabit these localities.

*Value of House Property.* The houses in the poor-law district of Canterbury are rated to the poor at their full probable annual net value, on which a shilling in the pound is levied four times in the year; this—including that upon the land within the boundary—produces the annual sum of £8,000. About 53 per cent. of the houses are rated at less than £5 a year each.

*Burial Grounds.* With the exception of those in the parishes of St. Dunstan's and St. Martin's, the parish burial grounds have lately been closed by an order in Council. To meet this event, and for the purpose of affording greater accommodation, a public cemetery of about four acres was purchased and consecrated about five years since; a small piece of ground of about half an acre, unconsecrated, has also been made available for the same purpose. These are said to be still capable, according to the present rate of mortality, of affording ample space for the next six or seven years. The municipal authorities, under a power invested in them, have now in contemplation either to purchase more ground contiguous to that already consecrated, or to establish another cemetery at the opposite side of the city.

*Population.* The population of Canterbury, according to the last census, was 18,398 inhabitants, giving an increase of 3,025 in the preceding twenty years. The rate of increase, however, is very uncertain, in consequence of the variable number of military stationed in the barracks, which, at the date of the last census, contained but a very small number, whereas in the two years reported upon in the tables they had been more than usually filled. There has evidently been so slight an increase in the resident population of the city, that it is impossible to estimate with any degree of exactness the actual population at any intermediate date. In reporting the comparative mortality to the number of persons living, it seems desirable to deduct the military in barracks and their mortality, and also the proportion of patients in the Kent and Canterbury Hospital not belonging to the city and their mortality; this will give us, according to the last census, a population of 17,958 resident inhabitants, and the average annual number of deaths 2·37 per cent. This, there is no doubt, represents a somewhat higher proportion than the reality; for, although in the years 1854 and 1855 there was more than the usual rate of mortality, exception should be made for the possible increase, however small, of the resident population, and also for the number of strangers usually attendant upon a full garrison, who are necessarily obliged to live out of the barracks, but cannot be separated from the resident population. This is the more manifest from the great excess which has occurred in the parishes of St. Mary's Northgate and St. Gregory's, which are the most contiguous to the barracks, and in which this class of persons mostly take up their temporary residence.

*Occupation.* The poorer classes of the city are mostly employed in agricultural pursuits; the wages for which, although but moderate in amount, are generally sufficient throughout the year to enable them to provide their families with the necessaries of life; in addition to which, the seasons of harvest and hop-picking oftentimes enable the families to lay by a little store against the requirements of winter.

*Proportion of Poor.* In consequence of certain parts of the city forming portions of two other poor-law unions, it has been impossible to form an estimate of the proportion of poor to the aggregate number of inhabitants in the entire city. There is reason, however, for supposing that, while the greater number of the population may be so estimated, these may be considered a fair criterion by which to judge of the whole.

That portion of the city included in the poor-law union of Canterbury comprises a population, according to the last census, of 14,100 inhabitants; of these, there were upon an average weekly in 1854, 119; and in 1855, 108 paupers, including 6 imbeciles or idiots, receiving in-door relief. In 1854 there were 439, and in 1855, 450 paupers, including 8 having medical relief only, and 24 non-resident paupers having out-door relief, as the weekly average. In addition to these, upon a weekly average, 6 lunatics were maintained in an asylum; and 27 vagrants, although not, strictly speaking, the poor of Canterbury, were upon a weekly average receiving temporary relief from its rates. Of these latter, in the year ending March 31st, 1856, 1,275 made application, and were relieved at the trifling cost of £5 : 11 : 10.

It has not been in my power to ascertain whether this number of paupers is large or small in proportion to the total number of inhabitants; but it is more probably the latter, inasmuch as, by referring to the return of expenses made to the assistant poor-law commissioner for the year ending Lady-day 1855, it appears that the weekly cost per cent. of population for maintaining the poor is less in Kent than in Cambridgeshire, Essex, Norfolk, or Suffolk, with which counties it is associated; and also that the cost per cent. of population in Canterbury is as nearly as possible the average of that in the entire county of Kent.

*(To be continued.)*

